# Second Lieutenants for the Medical Dept. of the Army

**Published:** 1918-05

**Authors:** 


**A Monthly Review of Medicine and Surgery**

EDITOR AND PUBLISHER, DR. A. L. BENEDICT, 228 Summer St., corner of Elmwood Ave., Buffalo, (Address for all communications. Please make personal and telephone calls before 1 P. M.)

Third, in age, on the western continent. Independent, but supports professional organizations. News limited mainly to Western N. Y. and Penn.

Subscription $2.00 a year; $3.00 for two years in advance. Changes and errors in address or failure or duplication of delivery, should be reported immediately.

Yearly Volume 73 MAY, 1918 Number 10

### Second Lieutenants for the Medical Dept. of the Army.

As in regard to the propaganda for universal military service, we feel that great care should be taken not to embarrass any branch of the federal government by urging any action, however much it may appeal to us, that might distract attention from the immediate emergency. The present is not the time to look too far ahead. The fact that a majority of our editorial staff is in the service of this country and our allies increases our reluctance even to appear to advise their superiors. However, the following may be set forth for what it is worth.

In June, approximately 2,000 young physicians, lacking interne training will be available for the military medical service and about as many more undergraduates will be available in an emergency, having about the same medical education as the graduates of a comparatively few years ago, indeed, lacking mainly the clinical and clinical laboratory refinements added to the medical course with the institution of the fourth year, though not necessarily actually placed in the curriculum of the fourth year. One of the troubles of the military service, whether viewed from the standpoint of the service or of the individual, is the difficulty of getting men of mature years to do the clerical and detail work which the army surgeon is expected to do but which the overwhelming majority of the present staff has, for many years in civil practice, especially at hospitals, been accustomed to leaving to internes and assistants. On the other hand, one of the difficulties which have been encountered in securing free volunteering of young medical men is the lack of the ideal place for them. True, the medical student or graduate lacking in all the requirements for commission as first lieutenant, whether volunteering or conscripted, may usually enter the medical department, and may usually find valuable and congenial training along the lines of his special interests, may indeed secure rather rapid promotion to non-commissioned grades and may ultimately, complete his medical course and matters may eventuate so that he can, if he desires, secure a commission as a surgeon.

But there is no denying that there is a wide difference between the man who has just succeeded in conforming to the requirements for a first lieutenant's commission and the one who lacks only a little of the undergraduate training, interneship or standing of college of graduation and who is serving in the medical department of the army as a private or non-commissioned officer. The difference is embarrassing to himself, sometimes to his superiors, it is not unreasonable to suppose that it detracts considerably from the number of men volunteering.

The present medical service needs men of this kind, not so much to spare the dignity of older men suddenly required to spend a considerable part of their time in rather rudimentary medical work, as to conserve for a higher grade of work, the energy lost to the government. On the other hand, in strictly clinical, laboratory work and even didactic instruction—for wherever medical men of the army are gathered in sufficient numbers, they are receiving actual instruction and not merely opportunities to observe cases—the army is now prepared to furnish to every young man in the medicine the equivalent of the fourth year of a college course, barring perhaps some technical points that could easily be supplied in a comparatively short special course to fit him thoroughly for graduation with the degree of M. D. It is also prepared to furnish an interneship far in advance of the average one available in private life.

Perhaps it is worth considering whether young men of the type mentioned, might not be commissioned second lieutenants and after a year's service as military internes and assistants, be turned back to their colleges for an intensive course to supply whatever deficiencies there might remain preliminary to the receipt of the degree of M. D., and after receiving the degree, be subject to promotion to the grade of first lieutenant on examination.

